# Integrated Amorphous Silicon p-i-n Temperature Sensor for CMOS Photonics

**DOI:** 10.3390/s16010067

**Published:** 2016-01-06

**Authors:** Sandro Rao, Giovanni Pangallo, Francesco Giuseppe Della Corte

**Affiliations:** Department of Information Engineering Infrastructures and Sustainable Energy (DIIES), “Mediterranea” University, Reggio Calabria 89122, Italy; giovanni.pangallo@unirc.it (G.P.); francesco.dellacorte@unirc.it (F.G.D.C.)

**Keywords:** p-i-n diode, temperature sensors, amorphous silicon, photonic integrated circuit

## Abstract

Hydrogenated amorphous silicon (a-Si:H) shows interesting optoelectronic and technological properties that make it suitable for the fabrication of passive and active micro-photonic devices, compatible moreover with standard microelectronic devices on a microchip. A temperature sensor based on a hydrogenated amorphous silicon p-i-n diode integrated in an optical waveguide for silicon photonics applications is presented here. The linear dependence of the voltage drop across the forward-biased diode on temperature, in a range from 30 °C up to 170 °C, has been used for thermal sensing. A high sensitivity of 11.9 mV/°C in the bias current range of 34–40 nA has been measured. The proposed device is particularly suitable for the continuous temperature monitoring of CMOS-compatible photonic integrated circuits, where the behavior of the on-chip active and passive devices are strongly dependent on their operating temperature.

## 1. Introduction

Si-based photonic devices have experienced tremendous progress in recent years [1,2,3] and a large scale fabrication of photonic and combined photonic-electronic integrated circuits (PEICs) show a strong uptrend in developing markets like datacom, telecommunication, and sensing [4,5,6,7,8,9].

Hydrogenated amorphous silicon (a-Si:H) is, in particular, a promising platform enabling the desired matching between electronics and on-chip photonics [10]. In a back-end approach, thin layers of a-Si:H can be in fact deposited on top of electronic microchips using the CMOS-compatible low-temperature plasma-enhanced chemical vapor deposition (LT-PECVD) technique, with no impact at all on the underlaying microelectronic circuit. a-Si:H can be also deposited on different substrates where crystalline silicon (c-Si) could not, be it a glass, a metal, an already processed silicon (Si) wafer, or even plastic.

However, many fabricated microelectronic or photonic integrated devices are very sensitive to external variations including temperature, bias voltage, and working wavelengths. In particular, Si-photonic active devices are strongly temperature-dependent, namely they are sensible to the environment temperature modifications due to the large thermo-optic (TO) coefficient of Si [11]. In the last decades, the refractive index temperature dependence has been modeled analytically and the Si TO coefficient has been related to the variation of the inter-band transition energies at some critical points of the Si band structure [12]. Moreover, it has been shown that the TO coefficient of a-Si:H is slightly higher than that of c-Si, resulting, respectively, *dn/dT* = 2.3 × 10^−4^ K^−1^ [13] and *dn/dT* = 1.8 × 10^−4^ K^−1^ [12] at the wavelength of 1550 nm at T = 300 K, which might diminish in part the advantages brought by the easy and low cost technology of a-Si:H.

As anticipated, in fact, refractive index variation with temperature can determine a non-correct behavior of integrated active devices such as interferometers, modulators, photodiodes, sensors. Therefore, thermal challenges need to be resolved in order to advance the silicon photonics to its final industrial stage. For example, the thermal sensitivity of the resonant wavelength for Si-based ring resonators is, e.g., approximately 100 pm/°C [14] or, to mention another example, in a Mach Zehnder (MZ) interferometer the TO effect is responsible of a wavelength shift of 90 pm/°C [15]. In Vernier effect-based photonic sensors, the microchip must be thermally stabilized to prevent the drift of the sensor output signal [16]. Consequently, the temperature-dependent optical performance of an on-chip integrated photonic device is a real issue to be taken into account during the design of PEICs [17], and these devices are not practical without a continuous thermal compensation during their operating life.

Recently, on-chip temperature measurement has been proposed for thermal variation compensation in many sensing devices such as humidity, pressure, flow, stress and gas concentration sensors [18].

Among different types of integrated temperature-sensing devices, the advantage of diode-based temperature sensors is the compatibility with IC technology, the low manufacturing costs, the quasi-linear output voltage-temperature, *V_D_-T*, behavior while preserving a high sensitivity [19].

When a diode operates as a temperature sensor, it is forward-biased at a current *I_D_* kept constant over the whole temperature working range and the corresponding voltage drop *V_D_* allows an accurate indirect temperature measurement. Low values of bias currents reduce device self-heating and the negative effects of the parasitic series resistance among which are a poorer linearity and sensitivity [20].

In the last years, few examples of a-Si:H p-i-n diode have been proposed as temperature sensor in a temperature range from *T* = 30 °C up to 90 °C. The devices showed a good linearity and a sensitivity of 3.2 mV/°C for a DC bias current of 10 nA [21].

In this work, a temperature sensor based on an a-Si:H p-i-n diode integrated in photonic layer is presented in detail. The sensor is placed very close to a MZ electro-optic modulator in order to monitor the chip temperature variations. The linear dependence of the voltage drop across the forward-biased diode on temperature from *T* = 30 °C up to 170 °C has been accurately measured.

## 2. Device Structure

**Figure 1:**
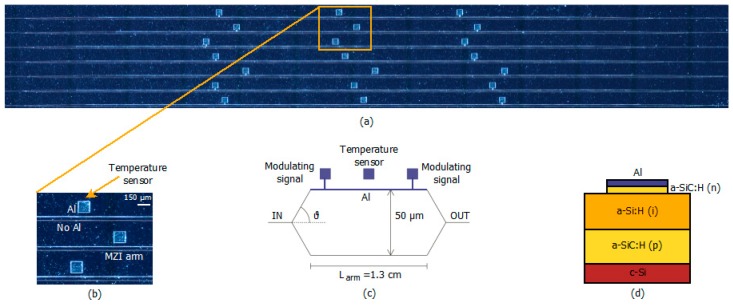
(**a**) An optical microscope image (top view) of the a-Si:H-based MZI modulators and temperature sensors; (**b**) temperature sensor detail; (**c**) schematic MZI and temperature sensor (plot not in scale); and (**d**) cross section of the integrated a-Si:H p-i-n diode temperature sensor.

The diode temperature sensor was integrated in the proximity to a Mach Zehnder interferometer (MZI), Figure 1a,b, and in particular close to the MZI arm where the propagating optical signal is phase shifted by carrier depletion induced in turn by the electric-field applied across the p-i-n diode. The schematic layout of the realized device is shown in Figure 1c together with its geometrical dimensions. More details about the MZI fabrication and operation are provided in [22,23].

The schematic cross section of the fabricated a-Si:H waveguide, integrating the vertical p-i-n diode is shown Figure 1d. It consists of an intrinsic a-Si:H layer, 2-μm-thick, between a p-doped a-SiC:H, 2-μm-thick, and an n-doped a-SiC:H, 300-nm-thick. The p-i-n cathode top contact is a 200-nm-thick Al layer. The active area of the device is 2.25 × 10^−4^ cm^2^.

As well-known, the *I_D_* current flowing in a p-i-n diode at a given applied voltage *V_D_* can be analytically described using the following formula:

(1) $$I_{D}=I_{S}\left(e^{\frac{qV_{D}}{\eta kT}}-1\right)$$

where *η* is the ideality factor, *I_s_* is the saturation current, *q* is the electric charge and *k* is the Boltzmann constant.

The characterization of the sensor output has been performed under forward bias condition where, at constant DC current, the voltage across the diode is linearly dependent on the temperature.

In fact, for *q∙V_D_>>* η*∙k∙T*, the voltage dependence on temperature can be obtained from Equation (1), yielding:

(2) $$V_{D}=\frac{kT}{q}\eta \ln\left(\frac{I_{D}}{I_{S}}\right)$$

Equation (2) makes explicit the linear dependence *V_D_-T* as long as the non-linear contribution of *I_s_* can be considered negligible with respect to *I_D_* [24,25].

## 3. Experimental Results and Discussion

**Figure 2:**
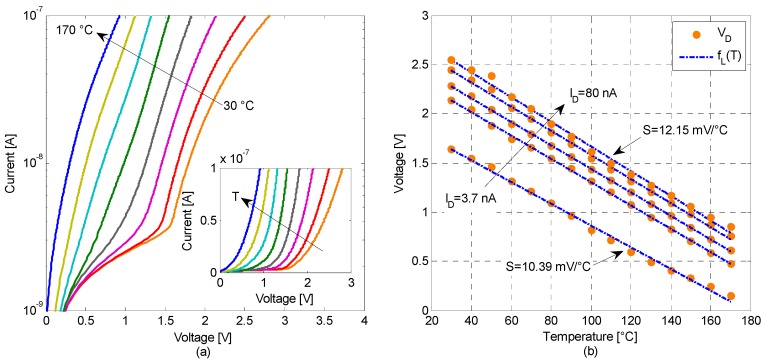
(**a**) Forward current-voltage characteristics for temperatures ranging from 30 °C up to 170 °C, with Δ*T* = 20 °C steps. The inset shows a detail of the *I_D_-V_D_* characteristics in linear scale. (**b**) Measured (points) forward voltages *versus* temperature at different bias currents (*I_D_* = 3.7, 22.8, 37.3, 60.4, 80 nA). Experimental data are fitted with the best-calculated linear model *f_L_(T)*.

In our setup, the p-i-n diodes were biased with a current *I_D_* kept constant in the whole temperature range. The devices were tested in a climatic chamber (Galli Genviro-030-C) setting the reference temperature through its internal PID digital microcontroller. A calibrated PT100 sensor, with an accuracy of ±0.3 °C, was placed in contact with the device under test in order to monitor, during the measurements, the exact temperature set points gradually varied from (to) 30 °C to (from) 170 °C. By using an Agilent 4155C semiconductor parameter analyzer, tests were made for *I_D_* varied in a range from 1 nA to 80 nA (±100 pA accuracy and 10 pA resolution, for generated currents in the range −100 nA < *I_D_* < 100 nA) and the corresponding voltage drop *V_D_* across the a-Si:H p-i-n diode was measured. In Figure 2a we report the *I_D_-V_D_* characteristics, for different temperatures in a range from 30 °C up to 170 °C.

From *I_D_-V_D_-T* measurements, a highly linear behavior of the voltage drop across the p-i-n diode on different temperatures was extracted, as shown in Figure 2b.

In our analysis, the coefficient of determination *(R^2^)* [26] has been calculated to evaluate the agreement between the experimental measurements and their linear best-fit, *f_L_(T)*. In particular, *R^2^* allowed us to quantify the sensor linearity goodness by fitting the experimental data with a linear model.

In the same figure, the measured data are fitted with the best-calculated linear model showing a good degree of linearity (*R^2^* > 0.995) for the whole considered range of *I_D_*, 3.7 nA to 80 nA.

The sensor sensitivity, *S*, is defined as the temperature derivative of Equation (2) and, therefore, it can be calculated from the slope of the *V_D_-T* characteristics.

As reported in Figure 2b, when *I_D_* is 3.7 nA the sensitivity is 10.39 mV/°C and increases up to 12.15 mV/°C for *I_D_* = 80 nA.

**Figure 3:**
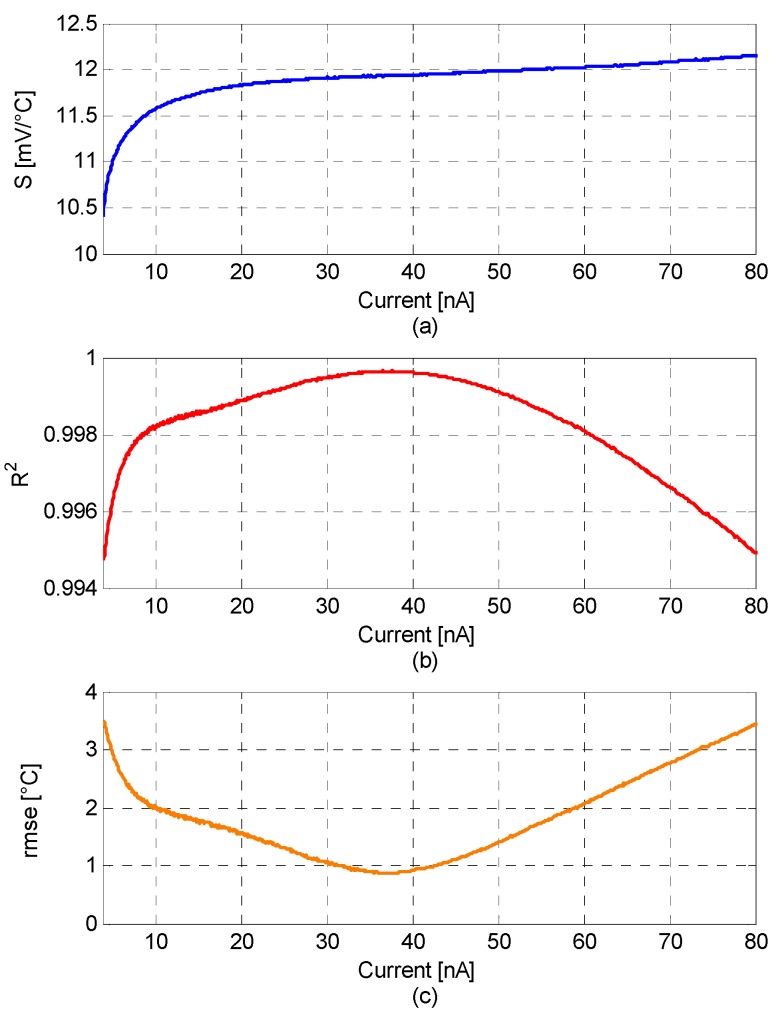
(**a**) Sensitivity, *S*; (**b**) coefficient of determination, *R^2^*; and (**c**) root mean square error, *rmse,* for the whole temperature range of 30–170 °C for bias currents between *I_D_* = 3.7–80 nA.

A more detailed analysis of *R^2^* and *S* is shown in Figure 3a,b for all values of *I_D_* in steps of 100 pA.

It is worth noting that the coefficient of determination varies by only 0.25% from an average of *R_a_^2^ =* 0.9972 over the considered temperature range leading to a temperature sensor with a highly linear behavior in a wide range of biasing currents. The maximum of *R^2^ =* 0.9996 occurs in the current range ≈ 34–40 nA. Within this current range, the sensitivity is constant, *S* = 11.93 mV/°C.

To evaluate the mismatch between the calculated linear best-fit, *f_L_(T)*, and the experimental measurements, the corresponding root mean square error *(rmse)* was first calculated and subsequently converted into a temperature error value using the following formula:

(3) $$rmse[{}^{\circ}C]=\frac{\sqrt{\frac{\sum_{i=1}^{n}{\left(V_{D}(T_{i})-f_{L}(T_{i})\right)}^{2}}{n}}}{S}$$

where *n* is the number of the temperature set points.

The calculated plot, *rmse versus I_D_*, for the considered temperature range is reported in Figure 3c. The temperature error is always lower than 3.5 °C, while the minimum *rmse* = 0.89 ± 0.01 °C is obtained for *I_D_* = 37.3 ± 3.3 nA.

It is worth noting that the current source accuracy of ±100 pA falls within the above reported range of *I_D_* for which the *rmse* and the sensitivity remain almost constant with no impact on the sensor performances. In practical applications, if we consider that the proposed device is intended for integration on a VLSI electronic microchip, the well-known technics for current stabilization based on bandgap references and current mirrors circuits could be used in association [27].

The temperature resolution, ε*_T_*, of a sensor measuring a temperature *T* is limited by the measurement system resolution, ε*_V_*, according to the expression:

(4) $$\varepsilon_{T}=\frac{\varepsilon_{V}}{S}$$

when the sensitivity of the sensor, *S = dV/dT,* does not change significantly within the considered temperature range. In our experimental setup, the measured voltage resolution is 20 µV leading to a theoretical device resolution of 1.7 m°C.

The large sensitivity allows in principle the resolution of small temperature variations, however, the smallest detectable temperature change is limited by several error sources including sensor and measurement system calibration, bias current and measured voltage accuracy, noise, sensor self-heating, *etc*.

The specific applications our integrated a-Si:H sensor is intended for, require however temperature measurements with an accuracy of the order of ±1 °C about.

In fact, if we consider, e.g., the wavelength division multiplexing (WDM) technique for fiber-optic communications, in which multiple optical signal channels are carried by a single fiber at different wavelengths of light, the spacing between two adjacent wavelengths is not smaller than Δλ~1 nm. In particular, for coarse wavelength division multiplexing (CWDM) is about Δλ = 10–20 nm, while in dense wavelength division multiplexing (DWDM) the spacing is about 0.8 to 2 nm [28]. Due to the tight spacing, DWDM systems require elaborate temperature control systems to ensure that the optical channels do not interfere each other.

As already mentioned, for actual Si-based integrated active photonic devices, the wavelength shift, due to the temperature change, does not exceed ~100 pm/°C [14,15], and therefore temperature measurements with resolution and accuracy of the order of a few tenths of a degree Celsius match the specifications of any practical control system.

The sensor precision, in term of stability and reproducibility, is a measure of how closely all the measured and calculated data are grouped around the characteristic mean values. In our analysis, the a-Si:H integrated temperature sensor was therefore accurately tested in order to evaluate how consistently it maintains a stable output over time by iteratively repeating three cycles of measurements, from (up to) 30 °C up to (from) 170 °C, in a long period of time and for five different diodes fabricated with the same process.

**Figure 4:**
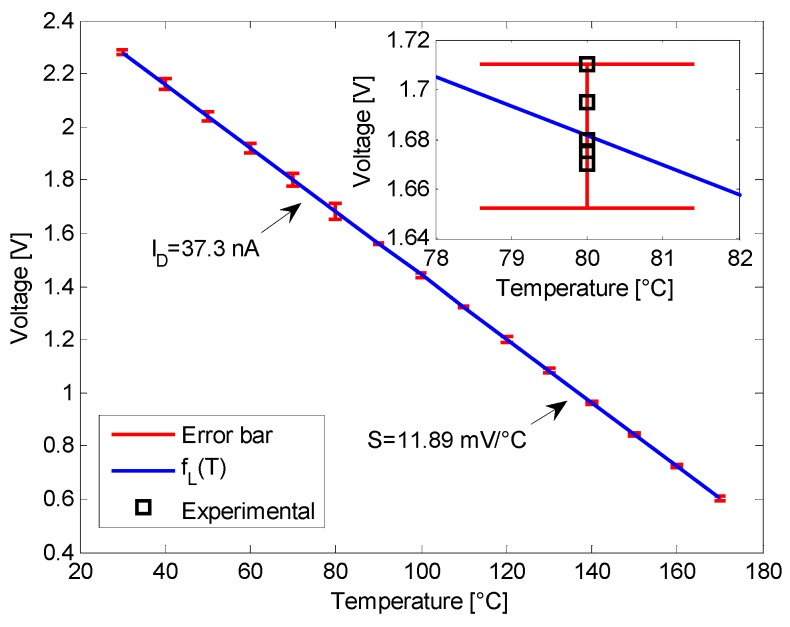
Linear fit and *rmse* of *V_D_ vs. T* for five different diodes fabricated with the same technological process and measured in a long period of time. The measurement cycles, from (up to) 30 °C up to (from) 170 °C, were done in different days. The bias current is *I_D_* = 37.3 nA for all five sensors. The inset shows the distribution of *V_D_* for the five different diodes at *T* = 80 °C.

The results are summarized in Figure 4, for *I_D_* = 37.3 nA, and leaded to a calculated maximum *rms* error lower than ±1.7%. Moreover, the coefficient of determination is *R^2^* = 0.9993 ± 3 × 10^−4^ and the corresponding sensitivity is *S* = 11.89 mV/°C with a standard deviation of 0.08 mV/°C.

In the inset of Figure 4, the measured voltage drops on the five characterized diodes are shown at the temperature of 80 °C, where the scattering is maximum.

**Figure 5:**
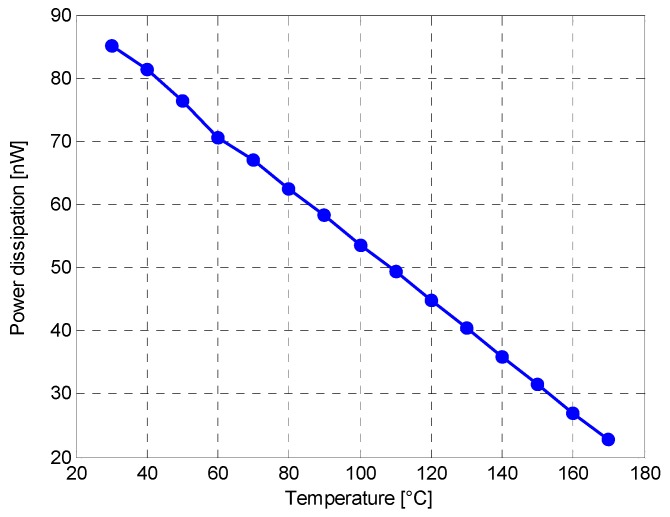
Power dissipation *vs.* temperature characteristics for *I_D_* = 37.3 nA.

In Figure 5 is shown the power dissipation (*P_D_*) of the a-Si:H temperature sensor, for *I_D_* = 37.3 nA, in the considered temperature range. The calculated values decrease with temperature with a maximum *P_D_* of ~85 nW at 30 °C, low enough to avoid device self-heating.

## 4. Conclusions

A high-performance temperature sensor based on a waveguide integrated a-Si:H p-i-n diode has been designed and characterized. The linear dependence of the voltage drop across the forward-biased diode on temperature, in a range from 30 °C up to 170 °C, was demonstrated.

Measurements showed both a high degree of linearity (*R^2^* = 0.9996) and a high sensitivity (*S* = 11.9 mV/°C) in the biasing current range ≈34–40 nA for a device area of 2.25 × 10^−4^ cm^2^.

Different cycles of measurements were iterated on five a-Si:H p-i-n diodes showing a long-term stability and good output repeatability.

The proposed a-Si:H temperature sensor, with low power dissipation, high sensitivity, and operating stability can be integrated into photonic integrated circuits (PICs) for sensing applications and inside CMOS compatible photonic active devices for which the temperature variation is a practical issue.
